# Magnetically switchable soft suction grippers

**DOI:** 10.1016/j.eml.2021.101263

**Published:** 2021-03-05

**Authors:** Anastasia Koivikko, Dirk-Michael Drotlef, Metin Sitti, Veikko Sariola

**Affiliations:** a Faculty of Medicine and Health Technology, Tampere University, 33720 Tampere, Finland; b Physical Intelligence Department, Max Planck Institute for Intelligent Systems, 70569 Stuttgart, Germany

**Keywords:** Soft gripper, Magnetorheological fluid, Hydraulic actuator, Suction

## Abstract

Grasping is one of the key tasks for robots. Gripping fragile and complex three-dimensional (3D) objects without applying excessive contact forces has been a challenge for traditional rigid robot grippers. To solve this challenge, soft robotic grippers have been recently proposed for applying small forces and for conforming to complex 3D object shapes passively and easily. However, rigid grippers are still able to exert larger forces, necessary for picking heavy objects. Therefore, in this study, we propose a magnetically switchable soft suction gripper (diameter: 20 mm) to be able to apply both small and large forces. The suction gripper is in its soft state during approach and attachment while it is switched to its rigid state during picking. Such stiffness switching is enabled by filling the soft suction cup with a magnetorheological fluid (MR fluid), which is switched between low-viscosity (soft) and high-viscosity (rigid) states using a strong magnetic field. We characterized the gripper by measuring the force required to pull the gripper from a smooth glass surface. The force was up to 90% larger when the magnetic field was applied (7.1 N vs. 3.8 N). We also demonstrated picking of curved, rough, and wet 3D objects, and thin and delicate films. The proposed stiffness-switchable gripper can also carry heavy objects and still be delicate while handling fragile objects, which is very beneficial for future potential industrial part pick-and-place applications.

## 1 Introduction

Grasping is one of the most important tasks for robots in manufacturing lines, food industry and warehouses. When manipulated objects are not fragile and their shape is well-defined, traditional rigid grippers perform well due to their ability to exert large forces and to maintain their shape during grasping. However, they struggle with fragile objects as they may damage the objects by applying too much local stress at the contact points. Furthermore, when the object shape is not well-defined (e.g. most biological objects), the grasping needs to be carefully planned, based on vision or otherwise, to account for variations in the object shape.

To overcome these challenges, soft robotic grippers [1] have been proposed. Being made of soft material, they can conform to the shape of the target object, distributing the stress more evenly. Multiple different soft grippers have been proposed: bioinspired suction cups [2,3], grippers with controlled adhesive surfaces [4–8], grippers with multiple soft fingers [9–13], grippers based on state change [14,15] and so-called jamming grippers [16–19], which envelope the target object with a switchable material that turns from soft to rigid (e.g. granular material [16,17] or magnetorheological fluids (MR fluids) [18–20]).

All of the aforementioned gripping methods have their short-comings: (1) suction cups and adhesive grippers generally exert smaller forces than rigid and jamming grippers; (2) jamming grippers can only pick objects that can be partially or completely enveloped by the gripper; and (3) adhesive grippers are mostly limited to dry and clean surfaces. To develop a gripping method without such short-comings, we decided to explore how the gripping forces of our existing soft suction cup gripper design [21] could be improved, without compromising its soft touch.

**Fig. 1:**
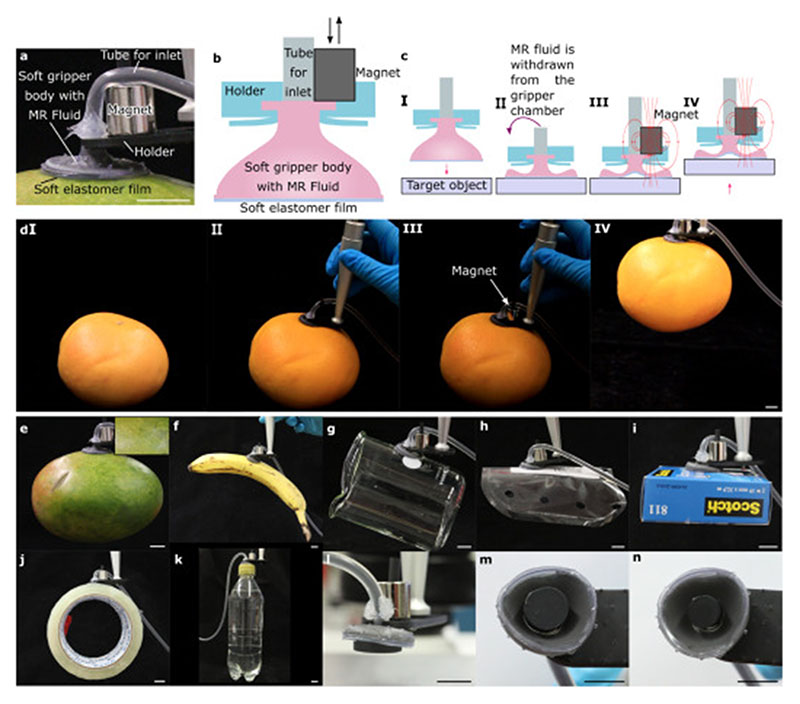
Schematic of the proposed stiffness-switchable gripper and its gripping demonstrations. (a) A photograph and (b) a schematic of the gripper. The soft gripper body is 3D-printed and attached onto a soft elastomer film. A silicone tube is attached to the inlet for the hydraulic connection. The gripper is filled with an MR fluid and attached to a 3D-printed holder including a removable magnet. (c) Schematic of the pick-and-place manipulation process and (d) corresponding photographs from a real experiment. (I) The gripper approaches the target object. (II) A preload is applied while withdrawing the MR fluid from the inside the gripper chamber. (III) A magnet is placed on top of the gripper for enhancing the pull-off force. IV) The gripper picks the object. Gripping demonstrations: (e) a mango (inset: a close-up of the surface of the mango), (f) a banana, (g) a wet beaker, (h) a thin plastic sheet, (i) a cardboard packet, (j) a tape roll, (k) a plastic water bottle, (l) ∅ 16 mm 3D-printed disc, (m) ∅ 10 mm 3D-printed disc and (n) ∅ 8 mm 3D-printed disc. Scale bars: 1 cm.

In this paper, we show that a soft suction cup gripper, filled with an MR fluid, can act as a switchable soft/hard gripper (Fig. 1a and b). The gripper consists of a 3D-printed bell-shaped gripper body, attached to a cast soft elastomer film (Fig. 1a and b). Between the body and the film is an internal cavity. At the top of the bell shaped body is an inlet, through which the cavity can be filled with an MR fluid. The inlet is connected to a syringe pump with silicone tubing. The basic gripper design is same as in our previous works [4,21]; however, unlike in our previous works, where the gripper cavity was filled with air, here the gripper is filled with an MR fluid, which enables the switchable stiffness.

During approach in Fig. 1c, I, the magnetic field is off, and the gripper is soft, conforming to the target object to get the best possible attachment without damaging the surface in Fig. 1c, II. Then, the magnetic field is applied in Fig. 1c, III; in this work by bringing a permanent magnet near the gripper. The magnetic field stiffens the MR fluid, so it does not move inside the gripper during the picking process in Fig. 1c, IV. The stiffness of the gripper prevents the object from peeling off and helps maintaining a good suction, effectively increasing the pull-off force. The entire gripping sequence is illustrated in Fig. 1c and d and demonstrations with varying objects in Fig. 1e–n.

MR fluids are often mineral or silicone oils containing micron sized iron particles. Under the external magnetic field, they show a reversible transformation from a liquid to a nearly solid-like state in less than milliseconds [22]. They are typically used in hydraulic dampers, brakes and control valves [22,23], but also as a semi-active actuation method in robotic grippers [24]. In soft grippers, they have been used by mixing them into silicones to create magnetic elastomers [25], by filling empty structures with them to create jamming effect based grippers [18–20], and in controllable wet adhesive grippers [26].

The main advantages of our new approach are: (1) the adhesion can be switched to apply large and small gripping forces as needed to grip heavy or fragile objects, respectively; (2) the softness of the gripper during approach allows it to conform to the shape of the object; (3) the gripper can pick both small and large objects (relative to its size); and (4) the gripper can adhere to many different surfaces, including curved, soft, rough, wet or oily ones.

## 2 Material and methods

### 2.1 Gripper design and fabrication

The gripper body was stereolithography printed (Form 2, Formlabs, Somerville, MA, USA) with an elastic resin (Elastic V1, Shore A 50). The body is a hollow bell-like structure, with an inner diameter of 20 mm, a height of 12.5 mm and a wall thickness of 700 μm. An inlet with a diameter of 4 mm is at the apex of the bell-like structure. To fabricate the soft elastomer film, we spread silicone elastomer (Ecoflex™ 00–50, Smooth-On Inc., Macungie, PA, USA) onto a polyethylene terephthalate (PET) sheet by using a universal film applicator (UA 3000, Mtv Messtechnik oHG, Germany). The thickness of the elastomer film after curing was ~400 μm.

To bond the film to the gripper, we cleaned the gripper with oxygen plasma (PICO with RF Generator, Diener electronic, 20 s at 30 W). Immediately after plasma treatment, we dipped the gripper in a silicone adhesive (Sil-Poxy™, Smooth-On Inc., Macungie, PA, USA) and pressed onto the cured elastomer film. The same silicone adhesive was used to attach a silicone tube (∅ 2/4 mm) to the inlet. Fig. S1 shows a schematic of the fabrication method.

### 2.2 Magnetorheological fluid

We tested filling the gripper with two different magnetorheological fluids (122EG and 140CG, Parker Lord, Macungie, PA, USA). The properties of the fluids are summarized in Table 1. To fill the gripper without having air bubbles, we first evacuated air from the gripper using an empty syringe, creating a near vacuum inside the gripper. Then, we swapped the syringe to one filled with an MR fluid and the vacuum was let to suck the fluid into the gripper cavity (Fig. S1). The total mass of the gripper with the fluid was approximately 9 g.

### 2.3 Characterization setup

A mechanical tester (TA.XT Plus, Stable Micro Systems, Surrey, UK) was used to characterize the gripping forces. The approach and retraction speeds of the tester were set to 0.1 mm/s. The gripper was attached to a 10 kg load cell, using a custom-made 3D-printed holder. The holder included a pole to which a ring-shaped neodymium magnet (grade N42, ∅ 5/12 mm, height: 12 mm, 4.4 kg holding power, weight: 8.5 g) could be placed. The center of the magnet was approximately 15 mm from the center of the gripper.

**Fig. 2:**
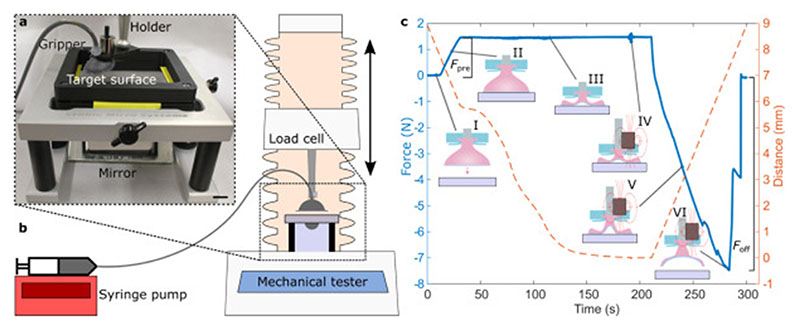
Pull-off force measurement method schematic and details. (a) Photograph of the experimental setup and (b) schematic. Experimental setup includes a syringe pump with a MR fluid and the gripper attached to a load cell of the mechanical tester. The target surface under the gripper is mounted to a holder containing a mirror for observing the attachment of the gripper during the experiment. Scale bar in the photograph: 1 cm. (c) An example of the measurement data. (I) The gripper approaches the target surface. (II) Once in contact, a preload force *F*_pre_ of 1.47 N is applied. (III) A volume *V* of the MR fluid is withdrawn while maintaining the preload. In this example, *V* = 1.5 ml and the fluid is 122EG. To maintain the preload while the gripper collapses, the controller decreases the distance as can be seen in the distance curve. (IV) The magnet is placed on top of the gripper. (V) The gripper retracts from the surface. (VI) The gripper comes off from the surface and the pull-off force *F*_off_ is measured. In this example, *F*_off_ = 7.47 N. The pull-off is not instantaneous but several peaks can be seen, due to parts of the film detaching at different times, until the gripper comes off completely.

The characterization experiments were done against a smooth and clean glass surface unless otherwise noted. A 45° mirror was mounted beneath the glass surface for observing the attachment of the soft film during the experiments. To control the MR fluid flow, a syringe pump (Aladdin Single-Syringe Pump, High Pressure, World Precision Instruments) was used. The pump was computer controlled over a serial port interface. The flowrate was 1 ml/min in all the experiments. Fig. 2a and b show the characterization setup.

**Fig. 3:**
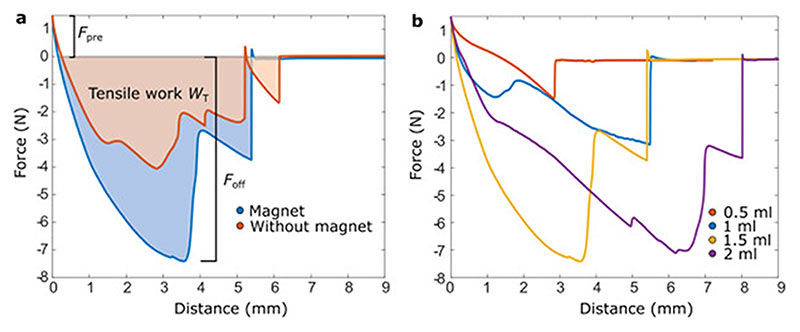
(a) Representative force–distance curves for the tensile part of the characterization experiment with and without the magnet. The retraction speed is 0.1 mm/s after constant preload *F*_pre_ of 1.47 N. The force needed for complete detachment is called *F*_off_ (here 4.05 N without magnetic field and 7.41 N with magnetic field applied). The area under the curve represents the tensile work *W*_T_ needed for detachment (here *W*_T_ = 13.65 mJ without magnetic field and 24.39 mJ with magnetic field applied). (b) Representative force–distance curves with different volumes *V* of fluid withdrawn (fluid:122EG). The magnetic field was applied in all these experiments.

Example of a typical characterization experiment is shown in Fig. 2c. First, the gripper approaches the target surface and once in contact, a preload force *F*_pre_ of 1.47 N is applied. Then, a volume *V* of the MR fluid is withdrawn from the gripper, which flattens the gripper body and creates vacuum under the film. A closed-loop controller maintains the preload during fluid withdrawal. After the withdrawal is finished, the external magnetic field is applied by placing the magnet on top of the gripper. The gripper then retracts and detaches from the surface. We call the peak force during the retraction as the pull-off force *F*_off_ and integral of the tensile part of the force–distance curve as the tensile work *W*_T_ (Fig. 3a).

## 3 Results and discussion

To confirm the effect of the magnetic field and suction on the gripping forces, we performed characterization experiments with and without the magnetic field applied and with varying volumes *V* of fluid withdrawn from the gripper. The representative force–distance curves from such experiments are shown in Fig. 3a and b.

The gripper film detached in multiple sudden jumps from the surface, which is seen as spikes in both Fig. 3a and b. The number and placement of the spikes varied between experiments, but they could be seen in almost all the experiments with and without external magnetic field applied. However, the overall shape of the curve, including the location and magnitude of the pull-off force peak, is quite comparable between the experiments, despite the smaller spikes seen in the experiments. The smaller spikes are due to parts of the film detaching at different times and vacuum readjusting under the gripper in a step-like manner. Another reason for the spikes is the step-like detachment of the gripper body from the soft film. When the MR fluid was withdrawn from the cavity, the gripper body collapsed onto the soft film. When the gripper started to retract from the surface, the gripper body detached from the soft film also in a step-like manner. The collapse of the gripper body onto the soft film is further illustrated in Fig. 5.

**Fig. 4:**
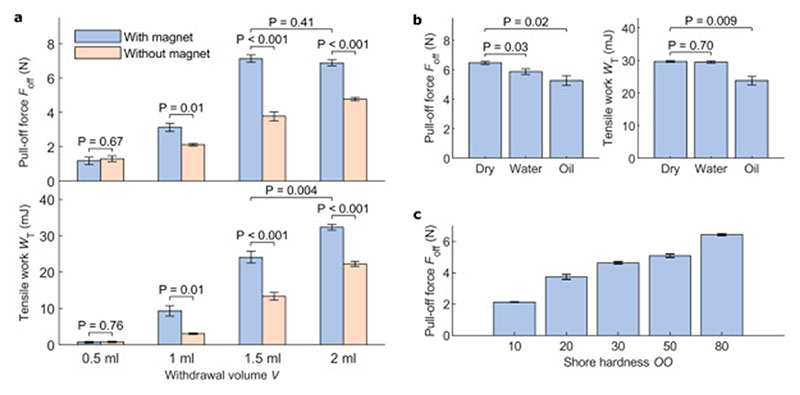
Characterization of the gripper. (a) Pull-off forces and tensile works for the gripper with different withdrawal volumes (fluid: 122EG). (b) Pull-off forces and tensile works for different surface conditions: dry, deionized water and heavy mineral (MR fluid: 122EG, V = 1.5 ml). (c) Pull-off forces for the gripper with objects with different softness. The error bars show standard error (n = 5) and the P-values are calculated using Welch’s unequal variances t-test.

The average pull-off forces and tensile works from five repeats with different combinations of experimental parameters are shown in Fig. 4a. The largest increase (90%, from 3.8 N to 7.1 N) in pull-off force with the magnet is seen when *V* = 1.5 ml. Note that, in the undeformed shape, the volume of the gripper chamber is ~1.5 ml, so *V* = 1.5 ml corresponds to 100% of the gripper chamber volume and majority of the MR fluid is already withdrawn from the chamber. When *V* was only 0.5 ml (33% of the chamber volume), the pull-off force was not observed to increase with the magnet. The pull-off forces increased with *V* until 1.5 ml, but no difference was seen in pull-off forces between 1.5 ml and 2 ml (133% of the gripper volume) when the magnetic field was applied. At such a large V, majority of the MR fluid is already withdrawn from the chamber and the extra withdrawn volume results in the compression of the silicone tubing connecting pump to the gripper, which is likely why the pull-off force was not observed to increase significantly after *V* = 1.5 ml. The tensile work was increased with the magnet for all tested withdrawal volumes from 1 ml and up. We conclude that, as long as a sufficient volume of fluid is withdrawn, the magnet can increase the adhesion significantly.

Many switchable adhesive grippers, such as electroadhesives and gecko inspired adhesives, fail to grasp surfaces wetted by water or oil [1]. However, most suction grippers do not suffer from this limitation [2]. To see how our gripper adheres to wet surfaces, we measured its adhesion against a glass surface wetted with deionized water or heavy mineral oil (CAS 8042-47-5, Sigma-Aldrich). The results are shown in Fig. 4b. The results show that the adhesion was largest to dry surfaces, and smallest to oily surfaces. However, the drop in pull-off forces was less than 20% between dry and oily surfaces. These results show that, due to merit of still working based on the vacuum principle, our gripper can adhere not only to dry surfaces, but also to wet and oily ones, unlike many previously reported switchable adhesive grippers that do not enclose the object.

Another challenge for switchable adhesive grippers can be gripping soft and deformable objects. To study our suction-based soft gripper’s performance in this challenge, we characterized how our gripper adheres to soft surfaces. We measured the maximum pull-off forces against ~0.7 cm thick flat objects having Shore-00 hardness ranging from 10 to 80. Note that these objects are still much softer than the gripper body (Shore ~A50). The objects were made by casting 20 g of elastomer into a petri dish, from which they were removed after being cured. Fig. 4c shows the results. The pull-off forces decreased as the Shore hardness decreased. Shore-00 30 corresponds approximately to human skin and even at this softness, the gripper was able to reach almost 5 N forces. We conclude that our gripper can pick also soft objects, although with reduced gripping forces, unlike many other switchable adhesive grippers [1].

The pull-off forces and tensile works are expected to vary with the MR fluid, due to different MR fluids having different properties, as documented in Table 1. To see the effect of MR fluid on the adhesion, we did the same characterization experiments for two different MR fluids, 122EG and 140CG. The representative curves are shown in Fig. S2. A gripper filled with 140CG fluid had much smaller pull-off force than a gripper filled with 122EG. The high viscosity and solid content of the 140CG fluid makes withdrawing it difficult: the fluid does not move smoothly inside the gripper during the withdrawal. With 140CG, all pull-off forces were observed to be less than 1 N. We conclude that the viscosity of the MR fluid is a highly critical parameter for successful gripping, because (a) the viscosity limits how quickly the fluid can be withdrawn from the cavity; and (b) high viscosity is related to high solid content, which can result in agglomeration of the magnetic particles at the inlet, clogging the inlet.

Rough surfaces can be challenging for adhesive grippers. To demonstrate that our gripper does not suffer from similar limitations, we picked up a red grapefruit (441 g, Fig. 1c) and a cardboard packet (44 g, Fig. 1i). The red grapefruit was rough (R_z_ = 17.7 μm, measured using an optical profilometer) with a visible pattern. These demonstrations show that the gripper can pick relatively heavy parts even with rough surfaces, which are typical for many everyday objects.

Fragile and thin objects can be difficult for jamming and vacuum grippers because the objects can break, or thin sheets can be sucked into the gripper. To demonstrate that our gripper can handle such objects, we picked up a mango fruit (418 g, R_z_ = 9.9 μm, Fig. 1e) and a thin plastic sheet (0.83 g, Fig. 1h). The gripper did not leave visible print onto the surface of the mango (Fig. 1e inset), even though the load was relatively large, and the surface was delicate and soft. Similarly, the plastic sheet did not show any visible marks, despite being thin.

Further gripping demonstrations included a banana fruit (143 g, Fig. 1f, uneven load/torque), a wetted glass beaker (116 g, Fig. 1g, a practical example of a wet surface), a tape roll (164 g, Fig. 1j, an example of a curved object), and a water bottle (518 g, Fig. 1k, a relatively heavy object). Taking all these demonstrations together, we conclude that the gripper is not highly specific to a particular material or surface type but can robustly pick many different types of everyday objects.

One potential drawback of suction cup grippers is that they might fail to pick objects smaller than the cup diameter, due to no vacuum forming between the film and the object. To see how our gripper fares with such objects, we tested picking discshaped objects with progressively smaller diameters (Fig. 1l–n). The smallest disc we could pick was ∅ 8 mm, which is already markedly smaller than the cup diameter (∅ 20 mm). These results show that the gripper cannot only pick objects larger than its diameter, but also objects slightly smaller than its diameter.

**Fig. 5:**
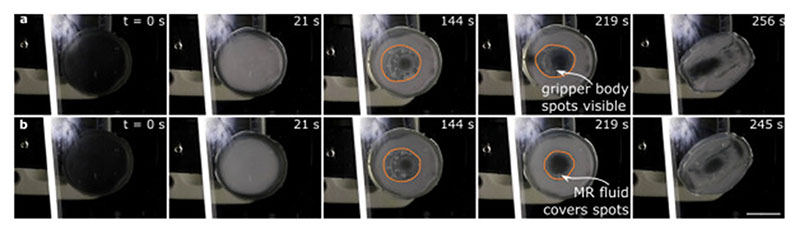
The behavior of the soft film of the 3D-printed gripper. Snapshots of the video showing the film of the gripper (a) under magnetic field and (b) without magnetic field. Orange circle indicates where the soft gripper body touches the soft film during the retraction. The body collapses against the film when the fluid is withdrawn. Due to the way the body is manufactured, it has small spots. The spots are clearly visible in some of the photographs. When the spots are visible, we know that there is a thin enough layer of MR fluid between the film and the body to allow the spots to be seen through the fluid. At the 219 s time point, there is a marked difference between (a) and (b): when the magnetic field is applied, the MR fluid does not flow and the body remains collapsed against the film, whereas the fluid can rearrange itself from the edges to the center when magnetic field is applied. Scale bar in all the photographs 1 cm.

To confirm that the magnetic field has an effect on the MR fluid inside the gripper and to observe how this affects the detachment of the film from the substrate, we recorded a video of the film detaching from the substrate with and without the external magnetic field applied. Video S1 and snapshots in Fig. 5 show the results. In the video, it can be observed that the fluid moves less when the magnetic field is applied (there is MR fluid in the middle of the gripper without external magnetic field applied). In the snapshots, this is most visible by comparing the snapshots from 219 s to the snapshots from 144 s: with the magnetic field is applied, the shape of the film and the fluid changes less than without the magnetic field. We take these results as an evidence that the magnetic field inhibits the movement of the fluid inside the gripper.

During all the experiments, we observed a few different ways the gripper can fail or break. The bonding between the film and the gripper body is a critical weak point and can tear (Fig. S3a) when excessive loads are gripped. In future, we aim to improve the film bonding by using different silicone adhesives and bonding surface treatments. Another failure mode is the sedimentation of the MR fluid in the silicone tube (Fig. S3b): if the gripper is unused for a few days, the magnetic particles start to sediment, separating the oil and the iron particles. This failure is not catastrophic, as the particles can be resuspended in the oil by repeatedly withdrawing the fluid from the cavity or by applying strong, varying external magnetic field. Finally, the silicone elastomers are air permeable and in a long period of time (~days), some air can be found inside the gripper cavity and the inlet tube. This failure can be remedied by refilling the gripper with the MR fluid.

Table 2 compares the performance of our gripper to some of the grippers previously reported in the literature. Shintake et al. [1] reviewed different soft robotic grippers and grouped different reported grippers whether they are based on actuation, controlled stiffness, or controlled adhesion. To our comparison table, we selected a few representative grippers belonging to each of these groups. For a more thorough review of the literature, we refer to the excellent review by Shintake et al. [1]. Compared to granular jamming and MR fluid jamming gripper, both stiffness-controlled grippers, our gripper can handle objects much heavier than its own mass. Also, unlike jamming grippers, our gripper does not need to completely enclose the target object or part of it. Jamming-based grippers are still faster than the current prototype of our gripper. Currently, the total time of the pick-and-place cycle of our gripper is limited by two different things: (1) it takes approximately 10 s for the syringe pump to withdraw the fluid (1.5 ml) from the cavity; and (2) manually placing the neodymium magnet to apply the magnetic field takes a few seconds. We do not think these are fundamental limitations of our gripper: faster pumps surely exist and MR fluids known to switch states less than in milliseconds [22]. A strong electromagnet could be used to quickly turn on the magnetic field. Typically, adhesion-controlled grippers (e.g., so-called gecko grippers) require the surface to be dry. There are attempts to enhance gecko-inspired adhesives so they adhere under water [27], but such adhesives require complex surface coatings which may degrade over time. Adhesive surfaces also struggle with contaminations, requiring careful consideration how to clean such surfaces to maintain their stickiness [28]. Our gripper does not require the surface to be dry and it is also very easy to clean our gripper, as the gripping surface is just a flat silicone film.

Finally, compared to commercial vacuum grippers, our gripper can handle a larger variety of objects: our proposed gripper, with its thin film, can pick thin films and objects somewhat smaller than its diameter. Wet/oily surfaces are also challenges to traditional open vacuum grippers, as liquids may contaminate the vacuum line. One advantage of our gripping method is that the film seals the fluidic line completely from the environment.

## 4 Conclusion

We have demonstrated a magnetically switchable soft suction gripper which can handle a wide range of fragile and complex 3D objects and surfaces. The switching mechanism is based on a magnetorheological fluid, which allows the gripper to stiffen under the presence of an external magnetic field. The gripper can handle over 7 N loads while the diameter of the gripper is only ~20 mm.

We envision that our gripper will be particularly useful for future packaging and warehouse applications, which typically involve gripping many different types of objects. The strength of our gripper in such applications is that it can pick rough, soft, wet, oily and curved surfaces and objects of various sizes. Another advantage of our gripper is that the gripping force can be carefully controlled: delicate light objects could be gripped with small suction and without the magnetic field, while heavy objects can use a combination of strong suction and magnetic field.

Another potential future application of our gripper is in industrial assembly. When soft, our gripper can conform to the shape of the object, allowing greater tolerances during picking and releasing. High precision tasks e.g. precision assembly will benefit from the gripper being in rigid state. Our proposed gripper design may also simplify the overall system complexity, by reducing the need for carefully measuring the object position before picking, or even by completely eliminating the need for visual feedback during picking.

Finally, our gripper is light-weight, relatively easy to fabricate, and can tolerate alignment errors during picking. These aspects suggest it will be feasible to use several of such grippers in parallel: multiple grippers picking multiple objects simultaneously. Every gripper would not need its own fluid line and magnet, but a single line and a magnet could be shared by several grippers. In parallel operation, our gripper has potential to greatly increase the throughput of industrial pick-and-place operations.

## Supplementary Material

Supplementary material related to this article can be found online at https://doi.org/10.1016/j.eml.2021.101263.

MMC S1

Video S2

## Figures and Tables

**Table 1:** Properties of the two MR fluids tested.

MR fluid property	122EG	140CG
Viscosity (mPa s)	42 ± 20	280 ± 70
Yield stress at 100 kA/m (kPa)	20	44
Density (g/cm^3^)	2.28–2.48	3.54–3.74
Solid content by weight (%)	72	85.44

**Table 2:** Comparison of few proposed gripping methods to fabricated gripper with MR fluid.

Gripper type	Ref.	Max. Preload (N)	Gripper diameter (mm)	Max. Pull-off force (N)	Max. lifting ratio^a^	Diameter ratio limits^b^	Surface conditions			Max. Surface roughness tested R_z_ (μm)	Time to acquire grip (s)	Power required to maintain grasp (W)
							Dry	Watery	Oily			
Granular jamming	[16]	150	86	100	?	0.1–0.85	Yes	?	?	?	0.1–1.1	^c^
MR fluid jamming	[20]	40	108	50	1.7	0.2–0.4	Yes	?	?	?	<0.1	75
Bioinspired suction gripper	[2]	0.5–1	9–14^d^	3.3	3.9	>2^e^	Yes	Yes	Yes	36.5	20^f^	?
Magnet-embedded suction cup	[29]	0.3	10	0.9	?	>3	Yes	Yes	Yes	?	140^g^	0
Same size commercial suction cup for uneven workpieces	[30]	?	20	11	>48	>1	Yes	Yes^h^	Yes^h^	?	<0.1^i^	c
Gecko-inspired gripper	[31]	?	180^j^	43	200	>0.5^k^	Yes	?	?	?	<0.1	0
**This work**		**1.5**	**20**	**7.5**	**80**	**>0.4**	**Yes**	**Yes**	**Yes**	**17.7**	**10**	**0**

a Object mass/gripper mass.

b Object diameter/gripper diameter.

c Power the vacuum unit needs.

d Diameter of a single suction cup.

e Estimated from Fig. 5 in [2].

f Estimated from the video in [2].

g Estimated from the video in [29].

h Separated filtering system for liquids is needed.

i Depends the vacuum system used.

j Estimated from the Fig. 1 in [30].

k Estimated from the Figures in [31].
