# Supplementary material for: Magnetically switchable soft suction grippers
Source: Extreme Mech Lett. Author manuscript; Available in PMC 2021 Apr 7. (PMC7610552; doi:10.1016/j.eml.2021.101263)
Supplement: MMC S1 [file EMS119934-supplement-MMC_S1.docx]

**Magnetically Switchable Soft Suction Grippers**

Anastasia Koivikko, Dirk-Michael Drotlef, Metin Sitti and Veikko Sariola

**Supplementary Material**


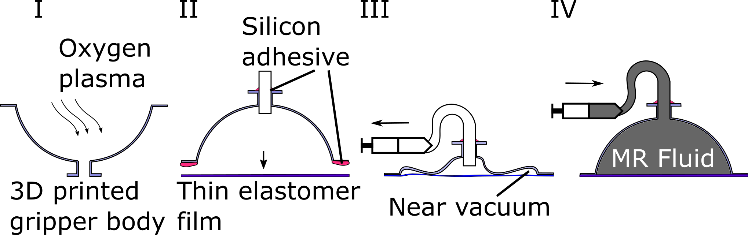


**Figure S1.** Fabrication and characterization of the gripper. a) Fabrication steps: I) 3D printed gripper body is cleaned with oxygen plasma and II) bonded by using silicone adhesive to the film and the inlet. III) The cavity is then evacuated by using an empty syringe and IV) filled with the MR fluid.


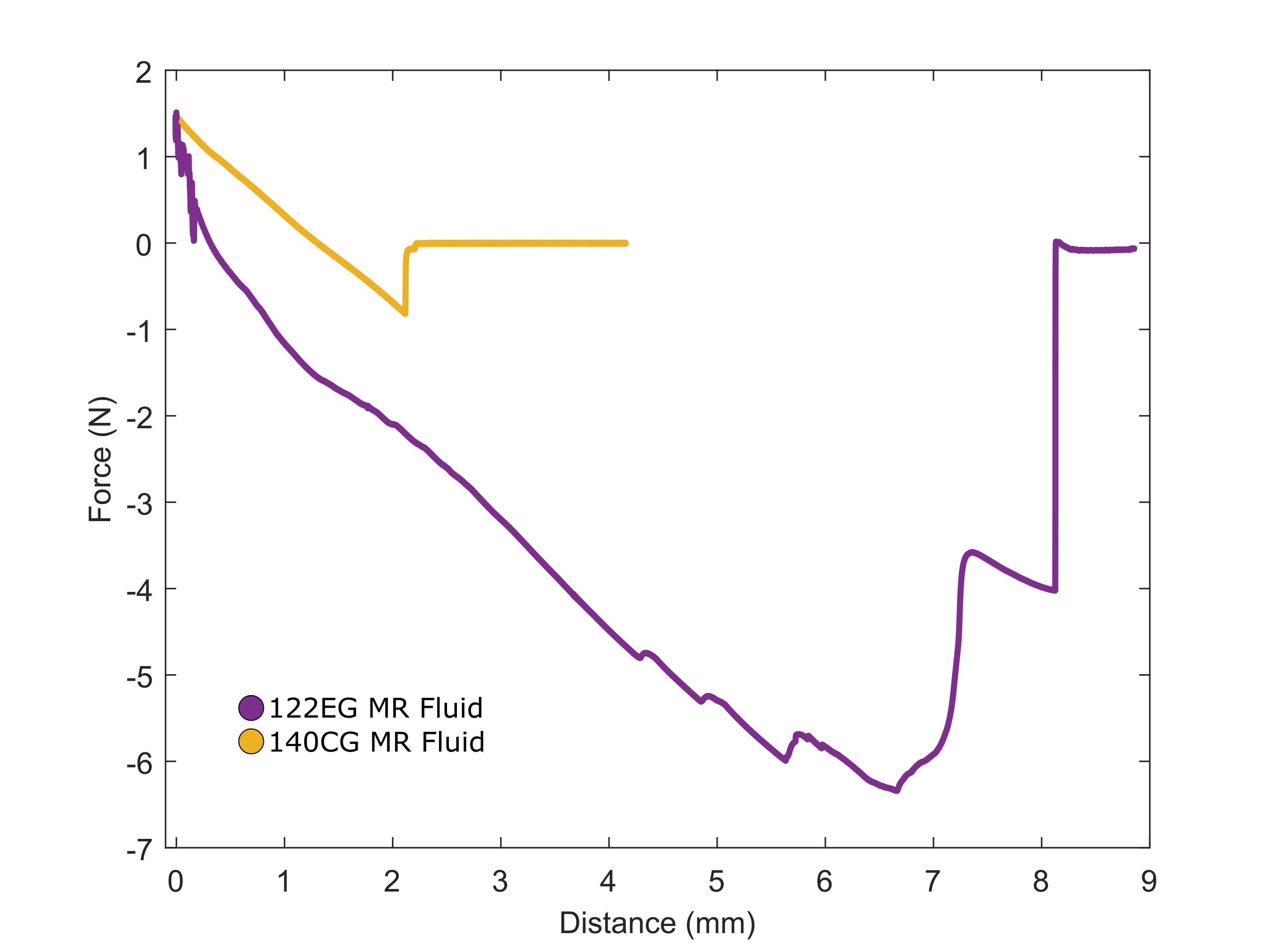


**Figure S2**. Force-distance curves for two different MR fluids (V = 1.5 ml).


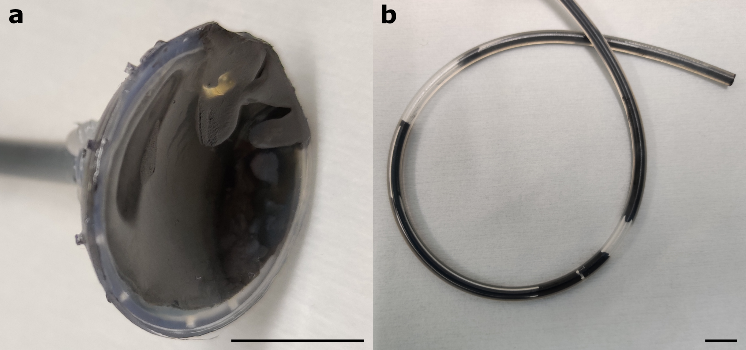


**Figure S3.** Possible failure modes of the gripper: a) Thin film tearing off the gripper body and b) air and sedimentation in the tube. Scale bar 1 cm.
